# Diet and Disease Development: Mechanisms, Prevention, and Treatment

**DOI:** 10.3390/nu16213676

**Published:** 2024-10-29

**Authors:** Sonia Saad, Hui Chen

**Affiliations:** 1 Department of Medicine, Kolling Institute of Medical Research, University of Sydney, Sydney, NSW 2065, Australia; 2 School of Life Sciences, The University of Technology Sydney, Sydney, NSW 2007, Australia

The overall aims of this Special Issue “Diet and Disease Development: Mechanisms, Prevention and Treatment” are to describe and emphasise the importance of diet in disease development; understand the mechanism(s) whereby an unhealthy diet can induce various diseases; examine the potential effects of natural compounds or extracts on disease prevention or treatment; and determine whether nutrients and their metabolites can be used as biomarkers to diagnose certain diseases. There are 10 articles published in this Issue, including 2 literature reviews. The topics covered include kidney disease, stroke, osteoporosis, fractures, dementia, fibrosis, sleep quality, constipation, and irritable bowel syndrome (IBD).

High fat diet and obesity are known to impose a significant risk of developing chronic kidney disease (CKD). One unit rise in body mass index (BMI) can increase CKD risk by 20% over 20 years [1]. Using an animal model of obesity induced by a combination of a high-fat and high-fructose diet, Chan and colleagues demonstrated that a long-term dietary folate deficiency can promote renal inflammation and subsequent renal fibrosis [2]. The authors also found a positive correlation between serum cholesterol level and kidney damage, regardless of whether they were induced by folate deficiency or a high-fat–high-fructose diet. Such findings highlight the importance of a healthy diet and adequate folate intake in maintaining immune regulation and renal function. Such findings are consistent with the research conducted by us and others, that high-fat diet consumption increases CKD progression in high-risk populations. Excess fat intake affects postprandial glycaemic control due to the interruption of the insulin signalling pathway and increases the risk of cardiovascular disease in patients with type 1 diabetes [3]. In the presence of additional insults (such as maternal obesity or maternal smoking), high-fat diet consumption also increases the risk of metabolic disease and CKD development [4,5].

In this Issue, we have demonstrated that a high-fat diet affects microbial diversity, promotes systemic inflammation, and causes the dysregulation of markers involved in lipid metabolism [6], inflammatory responses, oxidative stress, and renal fibrosis, particularly in the presence of nicotine-containing e-cigarette vapour inhalation [7]. Collectively, this emphasises the importance of an unhealthy diet in modulating disease progression and suggests that controlling the diet may reduce the risk of chronic disease.

Resveratrol is a potent bioactive chemical and natural antioxidant found in fruits and vegetables, particularly red wines, grapes, and blueberries. We and others have previously demonstrated that the antioxidant potential of resveratrol is largely mediated by sirtuin (SIRT)1. As such, resveratrol or SIRT1 over-expression can attenuate renal lipid accumulation and lipogenesis, oxidative stress, and inflammatory response, and ameliorate metabolic and kidney disease induced by high-fat diet consumption and maternal obesity [8,9,10,11,12]. In this Issue, Tain and colleagues investigated resveratrol butyrate esters (RBEs), which are novel derivatives of resveratrol produced following esterification with butyric acid, and their protective effects on the progress of hypertension and CKD [13]. They demonstrated that RBEs restored nitric oxide levels, reduced oxidative stress, balanced RAS levels and significantly prevented hypertension and kidney dysfunction in young rats with adenine-induced CKD. RBEs at a high dose demonstrated better benefits compared to low dose and resveratrol. This was associated with an increased abundance of the *Duncaniella, Ligilactobacillus,* and *Monoglobus* genera, as well as a reduction in *Eubacterium* and *Schaedierella*. To date, little is known about the genus *Schaedierella* and its role in disease development. Therefore, future research is required to better understand the role of microbiota in CKD pathogenesis and whether the same mechanism occurs in human patients with CKD and other kidney diseases. Safety studies are also required before recommending RBM as a dietary supplement in patients with CKD.

In addition to CKD, Yoshida et al. reviewed the literature to determine whether nutrient deficiencies affect other disease risks and whether nutrient intervention can prevent the development of or offer therapeutic benefits for these diseases [14]. In this review, the authors showed that malnutrition and certain nutrient deficiencies are not only associated with increased risks of CKD but with the development of other diseases, including stroke, osteoporosis, fractures, and dementia, and that nutritional intervention can reduce such risks and improve disease prognosis. Previous systematic reviews and meta-analyses also demonstrated the role of nutrients in the prevention and treatment of multiple diseases, including heart failure, CKD, diabetes mellitus, stroke, dementia, osteoporosis, fracture, as well as in sarcopenia and frailty [14]. Interestingly, the authors suggest that nutritional supplements or nutritional management, as well as patient education, can contribute to the complex and medical health issues seen in the aged population.

Subsequently, Guertler et al. completed a cross-sectional, controlled study of 296 patients with acne (120 patients) and rosacea (105 patients) with a matched control group to identify disease risk related to nutrition by recording dietary patterns over 4 weeks [15]. There were significant differences between the groups. A link between certain food types and acne or rosacea’s severity was discovered. As expected, chocolate, fried foods, and refined sugar were considered the top three triggers for acne, whereas alcohol, spices, and fried food were suggested as the top three triggers for rosacea. The adverse effects of these diets on acne and rosacea may be due to the presence of cinnamaldehyde, a compound that is widely used in food additives, which can trigger transient receptor potential ion channels on sensory nerves and keratinocytes. Consuming a healthy Mediterranean diet was beneficial for both skin conditions. Importantly, this study established dietary scores, which may serve as useful tools for assessing the risk of acne and rosacea based on dietary patterns. Further studies are required, however, to confirm the outcomes as dietary habits vary widely between different cultures and the diet effects and severity of symptoms were ranked subjectively by the patients. A more controlled study, conducted in a large cohort and different cultures over a longer period of time, is required to confirm the outcomes before clinical recommendations can be made for preventative measures and to promote long-term adherence to positive dietary changes.

Numerous bioactive substances and phytochemicals exert therapeutic effects and improve different diseases. α-mangostin, for instance, is isolated from mangosteen pericarp, which has been shown to possess anti-inflammatory, antioxidant and anti-cancer effects. Previous studies have demonstrated the potential role of α-mangostin in limiting pulmonary and liver fibrosis, as well as cardiac hypertrophy and fibrosis [16,17,18]. Its effect was mostly due to its role in suppressing myofibroblast differentiation and oxidative stress in fibroblasts. In this Issue, using primary fibroblast from the buccal mucosa tissues of healthy individuals or patients with oral submucous fibrosis, Lee et al. demonstrated that α-mangostin inhibits myofibroblast activities and fibrosis in patients with oral submucous fibrosis without affecting normal cells. This mechanism of action works by suppressing the TGF-β1/Smad2 signalling pathway and the expression of long non-coding RNA LincROR [19]. Such a finding supports the novel role of natural therapies, particularly α-mangostin, in oral fibrogenesis. Long non-coding RNA LincROR can reprogram pluripotent stem cells and suppress tumour protein p53 levels, which is induced after DNA damage [20,21]. LincROR also has a role in tumour progression and epithelial to mesenchymal transition and metastasis [22]. Whether α-mangostin has a role in oral cancer is unclear to date, which can be followed up in future studies. Having a natural nutritional supplement which can limit fibrosis and prevent or reduce cancer development or progression will have profound health impacts.

Multiple studies have demonstrated that psychobiotics can reduce stress and improve sleep quality. Bifidobacterium breve CCFM1025 (a psychobiotic strain) was previously shown to have a beneficial role in depression, Alzheimer’s disease, and other mental diseases. Lan et al. conducted a clinical trial in a small cohort of patients (40 patients) to assess the effect of CCFM1025 on stress-induced insomnia with a particular focus on the activity of the endocrine pathway [23]. Administration of CCFM1025 for 4 weeks was associated with changes in serum metabolites, including Daidzein, a phytoestrogen isoflavonoid. While this study provided strong evidence for psychobiotics in improving sleep quality, long-term multi-centre studies in larger cohorts are required to confirm their therapeutic effects, including research on their underlying mechanisms and potential side effects.

Wang et al. analysed the gut microbiota composition in 30 patients with functional constipation [24]. This study showed that Bacteroides and butyrate-producing bacteria were more abundant in patients with functional constipation and upstream products of host arginine biosynthesis were less abundant compared to healthy individuals [24]. This study needs to be validated in a large cohort before the outcomes can be generalised. Additional care needs to be taken to control for potential covariates. Nevertheless, this study supports the important role of microbiota in the development of functional constipation and provides new pieces of evidence which can be used as potential diagnostic markers for functional constipation and therapeutic targets.

Intestinal tryptophan (TRP) is an essential amino acid produced by ingesting lean meat, fish, dairy products, nuts, and seeds. TRP metabolism involves three main pathways in the gastrointestinal tract, including the kynurenine pathway, serotonin pathway, and gut microbiota indole pathway. These pathways are closely interconnected in affecting gut homeostasis. The regulatory role of different genes and metabolites within the tryptophan metabolism pathways in patients with IBD is largely unknown. In this Issue, Wang et al. completed a meta-analysis study on published transcriptomics datasets of intestinal biopsies of patients with IBD. A systematic review of metabolomics studies was also completed to identify how TRP and its metabolites change in stool, blood, and intestinal biopsies from patients with IBD [25]. They showed that the three pathways of intestinal TRP metabolism were involved in the pathogenesis of IBD and are closely interconnected, especially in active cases. The authors suggested that detecting TRP metabolites could potentially be used to identify patients with active intestinal inflammation. Changes in neuroactive metabolite production could explain the underlying mechanism of psychological distress related to IBD. The authors also suggest different strategies for IBD management through the regulation of TRP metabolism and identified gaps in the literature.

This Special Issue highlights the studies and reviews that contribute to new knowledge and offer expert opinions on the effects of dietary nutrients on disease prevention and development, as well as the underlying mechanisms of actions. It will inspire future research into related areas.

## References

[1] Than W.H., Chan G.C.-K., Ng J.K.-C., Szeto C.-C. (2020). The role of obesity on chronic kidney disease development, progression, and cardiovascular complications. Adv. Biomark. Sci. Technol..

[2] Chan C.-W., Lin B.-F. (2023). Folate Deficiency Enhanced Inflammation and Exacerbated Renal Fibrosis in High-Fat High-Fructose Diet-Fed Mice. Nutrients.

[3] Garonzi C., Forsander G., Maffeis C. (2021). Impact of Fat Intake on Blood Glucose Control and Cardiovascular Risk Factors in Children and Adolescents with Type 1 Diabetes. Nutrients.

[4] Glastras S.J., Chen H., Tsang M., Teh R., McGrath R.T., Zaky A., Chen J., Wong M.G., Pollock C.A., Saad S. (2017). The renal consequences of maternal obesity in offspring are overwhelmed by postnatal high fat diet. PLoS ONE.

[5] Huang T., Yang M., Zeng Y., Huang X., Wang N., Chen Y., Li P., Yuan J., Chen C., Oliver B.G. (2021). Maternal High Fat Diet Consumption Exaggerates Metabolic Disorders in Mice With Cigarette-Smoking Induced Intrauterine Undernutrition. Front. Nutr..

[6] Chen H., Burke C., Donovan C., Faiz A., Saad S., Oliver B.G. (2023). E-Cigarette Vapour Alters High-Fat Diet-Induced Systemic Inflammatory Responses but Has No Effect on High-Fat Diet-Induced Changes in Gut Microbiota. Nutrients.

[7] Feng M., Bai X., Thorpe A.E., Nguyen L.T., Wang M., Oliver B.G., Chou A.S.Y., Pollock C.A., Saad S., Chen H. (2023). Effect of E-Vaping on Kidney Health in Mice Consuming a High-Fat Diet. Nutrients.

[8] Nguyen L.T., Mak C.H., Chen H., Zaky A.A., Wong M.G., Pollock C.A., Saad S. (2019). SIRT1 Attenuates Kidney Disorders in Male Offspring Due to Maternal High-Fat Diet. Nutrients.

[9] Nguyen L.T., Chen H., Mak C., Zaky A., Pollock C., Saad S. (2018). SRT1720 attenuates obesity and insulin resistance but not liver damage in the offspring due to maternal and postnatal high-fat diet consumption. Am. J. Physiol. Endocrinol. Metab..

[10] Chen H., Pollock C., Saad S. (2017). SIRT1 reduction is associated with sex-specific dysregulation of renal lipid metabolism and stress responses in offspring by maternal high-fat diet. Sci. Rep..

[11] Gan Y., Tao S., Cao D., Xie H., Zeng Q. (2017). Protection of resveratrol on acute kidney injury in septic rats. Hum. Exp. Toxicol..

[12] Gong L., Guo S., Zou Z. (2020). Resveratrol ameliorates metabolic disorders and insulin resistance in high-fat diet-fed mice. Life Sci..

[13] Tain Y.-L., Chang C.-I., Hou C.-Y., Chang-Chien G.-P., Lin S., Hsu C.-N. (2023). Dietary Resveratrol Butyrate Monoester Supplement Improves Hypertension and Kidney Dysfunction in a Young Rat Chronic Kidney Disease Model. Nutrients.

[14] Yoshida S., Shiraishi R., Nakayama Y., Taira Y. (2023). Can Nutrition Contribute to a Reduction in Sarcopenia, Frailty, and Comorbidities in a Super-Aged Society?. Nutrients.

[15] Guertler A., Volsky A., Eijkenboom Q., Fiedler T., French L.E., Reinholz M. (2023). Dietary Patterns in Acne and Rosacea Patients—A Controlled Study and Comprehensive Analysis. Nutrients.

[16] Soetikno V., Murwantara A., Andini P., Charlie F., Lazarus G., Louisa M., Arozal W. (2020). Alpha-Mangostin Improves Cardiac Hypertrophy and Fibrosis and Associated Biochemical Parameters in High-Fat/High-Glucose Diet and Low-Dose Streptozotocin Injection-Induced Type 2 Diabetic Rats. J. Exp. Pharmacol..

[17] Tangphokhanon W., Pradidarcheep W., Lametschwandtner A. (2021). α-mangostin preserves hepatic microvascular architecture in fibrotic rats as shown by scanning electron microscopy of vascular corrosion casts. Biomed. Rep..

[18] Liao Y.J., Lee C.Y., Twu Y.C., Suk F.M., Lai T.C., Chang Y.C., Lai Y.C., Yuan J.W., Jhuang H.M., Jian H.R. (2023). Isolation and Biological Evaluation of Alfa-Mangostin as Potential Therapeutic Agents against Liver Fibrosis. Bioengineering.

[19] Lee Y.-H., Hsieh P.-L., Chao S.-C., Liao Y.-W., Liu C.-M., Yu C.-C. (2023). α-Mangostin Inhibits the Activation of Myofibroblasts via Downregulation of Linc-ROR-Mediated TGFB1/Smad Signaling. Nutrients.

[20] Jain A.K. (2020). Emerging roles of long non-coding RNAs in the p53 network. RNA Biol..

[21] Ghafouri-Fard S., Pourtavakoli A., Hussen B.M., Taheri M., Kiani A. (2023). A review on the importance of LINC-ROR in human disorders. Pathol.-Res. Pract..

[22] Liu M., Zhang M., Yin H. (2022). Linc-ROR promotes invasion and metastasis of gastric cancer by activating epithelial-mesenchymal transition. Indian J. Pathol. Microbiol..

[23] Lan Y., Lu J., Qiao G., Mao X., Zhao J., Wang G., Tian P., Chen W. (2023). Bifidobacterium breve CCFM1025 Improves Sleep Quality via Regulating the Activity of the HPA Axis: A Randomized Clinical Trial. Nutrients.

[24] Wang J., Wang L., Yu Q., Tang N., Mei C., Zhang H., Wang G., Lu J., Chen W. (2023). Characteristics of the Gut Microbiome and Serum Metabolome in Patients with Functional Constipation. Nutrients.

[25] Wang S., van Schooten F.-J., Jin H., Jonkers D., Godschalk R. (2023). The Involvement of Intestinal Tryptophan Metabolism in Inflammatory Bowel Disease Identified by a Meta-Analysis of the Transcriptome and a Systematic Review of the Metabolome. Nutrients.

